# NADPH Oxidase and the Degeneration of Dopaminergic Neurons in Parkinsonian Mice

**DOI:** 10.1155/2013/157857

**Published:** 2013-11-28

**Authors:** Marina S. Hernandes, Cecília C. Café-Mendes, Luiz R. G. Britto

**Affiliations:** Department of Physiology and Biophysics, Institute of Biomedical Sciences, University of São Paulo, São Paulo, 05508-900 SP, Brazil

## Abstract

Several lines of investigation have implicated oxidative stress in Parkinson's disease (PD) pathogenesis, but the mechanisms involved are still unclear. In this study, we characterized the involvement of NADPH oxidase (Nox), a multisubunit enzyme that catalyzes the reduction of oxygen, in the 6-hydroxydopamine- (6-OHDA-) induced PD mice model and compared for the first time the effects of this neurotoxin in mice lacking gp91^phox−/−^, the catalytic subunit of Nox2, and pharmacological inhibition of Nox with apocynin. Six-OHDA induced increased protein expression of p47^phox^, a Nox subunit, in striatum. gp91^phox−/−^ mice appear to be completely protected from dopaminergic cell loss, whereas the apocynin treatment conferred only a limited neuroprotection. Wt mice treated with apocynin and gp91^phox−/−^ mice both exhibited ameliorated apomorphine-induced rotational behavior. The microglial activation observed within the striatum and the substantia nigra pars compacta (SNpc) of 6-OHDA-injected Wt mice was prevented by apocynin treatment and was not detected in gp91^phox−/−^ mice. Apocynin was not able to attenuate astrocyte activation in SN. The results support a role for Nox2 in the 6-OHDA-induced degeneration of dopaminergic neurons and glial cell activation in the nigrostriatal pathway and reveal that no comparable 6-OHDA effects were observed between apocynin-treated and gp91^phox−/−^ mice groups.

## 1. Introduction

Parkinson's disease (PD) is characterized by a progressive loss of dopaminergic neurons in the nigrostriatal pathway of the brain, which triggers complex functional modifications within the basal ganglia circuitry. The decline of dopamine in the striatum is clinically associated with the typical motor symptoms of the disease, such as bradykinesia, tremor, rigidity, and postural instability [1, 2].

Although the etiology of PD is unknown, a common element of most theories is the involvement of oxidative stress, either as a primary or secondary event of the disease [2, 3]. Indeed, analysis of postmortem human brain tissue obtained from PD patients revealed elevated levels of the oxidative stress products such as 4-hydroxynonenal [4], protein carbonyls [5, 6], and 3-nitrotyrosine [7, 8].

Research on the pathogenesis of PD has rapidly advanced due to the development of animal models, which have largely contributed to the understanding of the progression of PD and to the development of potential therapeutic strategies [9, 10]. Although several toxin-induced animal models simulate the motor deficits occurring in PD, 6-hydroxydopamine (6-OHDA) lesions have been the most widely used animal models of PD since the 1970s, after the demonstration that the injection of this agent into the substantia nigra pars compacta (SNpc) was able to cause anterograde degeneration of the nigrostriatal dopaminergic system [11]. Ever since, the 6-OHDA model has been accepted as a valuable tool for replicating the PD-like loss of dopaminergic neurons in the SNpc [10]. The biological effects of 6-OHDA were considered to be mainly related to the massive oxidative stress caused by the toxin that, once accumulated in the cytosol, seems to be autooxidated, promoting a high rate of free radical generation [9]. However, an *in vitro* study using apocynin, a nonspecific pharmacological inhibitor of NADPH oxidase (Nox), suggested that Nox-derived reactive oxygen species (ROS) might be also involved in the 6-OHDA-induced dopaminergic degeneration [12].

Noxes are multisubunit enzymes able to transfer electrons across biological membranes, reducing molecular oxygen to superoxide at the expense of NADPH. The prototype Nox, the Nox2 isoform, is composed of the membrane subunits gp91^phox^ and p22^phox^ and of cytosolic subunits p47^phox^, p67^phox^, p40^phox^, and the small GTPase Rac. Nox2 is activated by forming a complex with its cytosolic activators. Upon activation, p47^phox^ becomes phosphorylated and the entire cytosolic complex translocates to the membrane, assembling with gp91^phox^ and p22^phox^, thus forming a functional Nox complex capable of reducing oxygen to superoxide [13]. It has been demonstrated that forty-eight hours after intrastriatal 6-OHDA injection, gp91^phox^ and p47^phox^ mRNAs were found to be increased in the rat ventral midbrain and striatum. There are demonstrations that Nox subunits are expressed in dopaminergic neurons in rat primary mesencephalic cultures and, most importantly, that the dopaminergic degeneration induced by 6-OHDA was significantly reduced by the treatment with apocynin [12].

Apocynin is a natural organic compound widely used as a Nox inhibitor. It is thought to prevent the translocation of cytosolic subunits to the membrane bound subunit gp91^phox^, thus inhibiting the activation of the enzymatic complex and, consequently, the superoxide production [14]. Despite the positive effects of apocynin in experimental PD studies, its mechanism of action is still controversial. Furthermore, apocynin has multiple side effects, altering the cellular glutathione levels and interfering with many cellular signaling cascades [15]. In light of those facts, the main purpose of this study was to characterize the involvement of Nox2 in the 6-OHDA-induced PD mouse model by comparing the effects of that neurotoxin on mice lacking gp91^phox^, the catalytic subunit of Nox2, and the pharmacological inhibition of Nox with apocynin. Behavioral testing, immunohistochemistry, and Western blotting assays were combined in order to evaluate that issue.

## 2. Materials and Methods

### 2.1. Animals

Ten-week-old male gp91^phox−/−^ mice (Jackson Laboratories, Maine, USA), along with wild type (Wt) mice (C57BL/6) weighing between 25 and 30 g, were used throughout this study. The animals had free access to food and water and were maintained on a 12-12 h light-dark cycle. Experiments were performed with age- and weight-matched animals. All procedures were approved by the Institutional Animal Care Committee of the Institute of Biomedical Sciences, University of São Paulo, Brazil.

### 2.2. Surgical Procedures

In order to lesion the nigrostriatal system, 6-OHDA was unilaterally injected into the right striatum of both gp91^phox−/−^ and Wt mice. The animals were anaesthetized using 2, 2, 2-tribromoethanol (2%, Sigma-Aldrich Co., St. Louis, MO, USA) and placed into a stereotaxic frame with nose and ear bars especially designed for mice. six-OHDA (Sigma Chemical Co., St. Louis, MO, USA) was dissolved at a concentration of 10 *μ*g/*μ*L in saline (NaCl 0.9%) with 0.1% ascorbic acid [16]. The injection was performed using a Hamilton syringe (model 701) at the following coordinates: AP: −0.4 mm; ML: ±2.0 mm; DV: −3.0 mm relative to the bregma [17]. The total volume injected was 1 *μ*L. The injection was conducted at a rate of 0.5 *μ*L/min and the needle was left in place for additional 3 min before it was slowly removed. The left striatum received 1 *μ*L of vehicle (saline in 0.1% ascorbic acid) in the same coordinates and was used as a control. Additionally, sham-operated mice were infused with 1 *μ*L of vehicle into both right and left striatum and served as controls in the apomorphine-induced rotation test. Clinical signs were also monitored daily after the surgery, including general body condition and dehydration. Behavioral analyses were typically conducted during the morning hours.

Animals used for immunohistochemistry to detect glial cell markers (*n* = 23) and for immunoblotting (*n* = 8) were euthanized for analysis 15 days after the surgery. An additional group of mice was subjected to the apomorphine-induced rotation test (*n* = 27) and their brains were used for immunohistochemical assays to detect tyrosine hydroxylase (TH).

### 2.3. Apomorphine-Induced Rotation Test

Apomorphine (Tocris Bioscience, Ellisville, MO, USA) was injected i.p. at a dose of 0.1 mg/kg [18]. Mice were placed in an automated rotameter (Rota-count, Columbus Instruments, Columbus, OH, USA) and allowed to adapt to their environment for 5 min before the rotations were recorded over 10 min. Results were expressed as number of rotations to the side contralateral to the lesion per minute.

### 2.4. Apocynin Treatment

Wt mice were treated with apocynin (200 mg/kg/day in the drinking water; [19] for 15 days after 6-OHDA injections).

### 2.5. Immunoblotting

For subcellular fractionation (membrane and cytosolic protein fractions), mesencephalic and striatal samples were quickly collected, frozen in liquid nitrogen, and stored at −70°C until use. Briefly, tissue proteins were isolated in 50 mM Tris HCl, 150 mM NaCl, 2 mM EDTA, 0.5% sodium deoxycholate, 2 mM sodium fluoride, 1% SDS, 1% Nonidet P-40, and protease inhibitors (Sigma-Aldrich Co., St. Louis, MO, USA). Homogenates were centrifuged for 10 min at 2,000  ×g at 4°C. The supernatants were collected and submitted to ultracentrifugation for 1 h at 100,000 ×g at 4°C. The resulting supernatants and pellets were designated as the cytosolic and membrane fractions, respectively. The protein concentration was determined using the Bradford method (Bio-Rad, CA, USA) and 10% acrylamide SDS gels (Bio-Rad, CA, USA) were loaded with 30 *μ*g of protein per lane. These proteins were then electrotransferred to nitrocellulose membranes (Millipore, Billerica, MA, USA) at 100 V for 80 min using a Trans-Blot cell. The membranes were then blocked for 2 hours at room temperature with phosphate buffered saline (PBS) containing 0.05% Tween-20 (TTBS) and 5% nonfat milk and incubated overnight at 4°C with anti-47^phox^ (Chemicon, USA) diluted 1 : 1000 in TTBS with 1% nonfat milk. The probed proteins were developed by using a chemiluminescent kit (ECL, Amersham Biosciences, NJ, EUA) and the bound antibodies were visualized using radiographic films. Loading controls were evaluated by antiactin (1 : 1000 - Sigma, EUA) blotting when cellular extracts were analyzed, and by anti-integrin (1 : 2000 - Abcam, EUA) when membrane fractions were analyzed. The quantification of band intensity was performed with ImageJ (National Institutes of Health, USA).

### 2.6. Immunoperoxidase

Mice were deeply anesthetized with ketamine hydrochloride (100 mg/kg of body weight, i.m.) and xylazine (16 mg/kg of body weight, i.m.) and subjected to transcardiac perfusion, with a buffered saline solution, followed by a fixative solution containing 4% paraformaldehyde (PFA) dissolved in 0.1 M phosphate buffer (PB, pH 7.4). The brains were collected, postfixed in PFA for 4 h, and transferred to a 30% sucrose solution in PB to ensure cryoprotection, which lasted for 48 h. Brain sections were obtained on a sliding microtome adapted for cryosectioning. The 30 *μ*m-thick coronal sections were collected in PB and arranged in 6 similar sets. Sections were incubated for 12–16 h with anti-OX42 (CD11b/c, Biosciences, CA, USA), anti-GFAP (Immunon, Pittsburgh, PA, USA), and anti-TH (Chemicon, Temecula, CA, USA) diluted 1 : 1000 in 0.3% of Triton X-100, containing 0.05% normal donkey serum. Following 3 washes of 10 min each with PB, sections were incubated for 2 h with a biotinylated secondary antibody (donkey anti-mouse IgG, Jackson ImmunoResearch, PA,USA, 1 : 200), then with the avidin-biotin complex (1 : 100; ABC Elite kit, Vector Labs, Burlingame, CA, USA). After washing, the sections reacted with 0.05% 3,3-diaminobenzidine and 0.01% hydrogen peroxide in PB. Intensification was conducted with 0.05% osmium tetroxide in water. The sections were mounted on gelatinized slides, dehydrated, cleared, and coverslipped. Controls for immunostaining included the omission of the primary antibody and its substitution for normal goat serum, which completely eliminated staining.

### 2.7. Glial Cell Image Acquisition and Quantification

Digital images were acquired for both striatum and SN on a light microscope. Each slide was scanned at 20x magnification. A total of eight sampling areas from the control and experimental striatum and SN from eight animals were acquired for analysis. Semiquantitative skeleton analysis method was performed using ImageJ software [20]. For skeleton analysis, the image threshold was adjusted to exclude background labeling and to visualize all glial cells and processes (OX42 and GFAP immunostaining); thresholding values were kept constant between matching control and experimental regions. The resulting image was converted to a binary, skeletonized and evaluated in terms of optical density. The results for each one of the experimental groups (Wt, gp91phox^−/−^ and Wt treated with apocynin) were then averaged and subjected to statistical analysis using GraphPad Prism 3.02 (GraphPad Software Inc., San Diego, CA, USA).

### 2.8. Immunofluorescence

In order to determine the expression of p47^phox^ in specific cell types, double-labeling experiments were carried out using purified antibodies designed to label neurons (NeuN), astrocytes (glial fibrillary acidic protein, GFAP), and microglial cells (OX42). Immunofluorescence tissue preparation was performed in the same conditions as in the immunoperoxidase assays, described above. Briefly, the brain sections were incubated free floating for 12–16 h with the following primary antibodies: mouse anti-rat GFAP (1 : 1000 - Millipore, Billerica, MA, USA), mouse anti-rat OX42 (1 : 1000 - Abcam, Cambridge, UK), mouse anti-rat NeuN (1 : 1000 - Chemicon, Billerica, MA, USA), and mouse anti-rabbit p47^phox^ (1 : 250 - Chemicon, Billerica, MA, USA) diluted in 0.3% of Triton X-100. Anti-rabbit FITC conjugated (1 : 250; Abcam, Cambridge, UK) and anti-mouse TRITC conjugated (1 : 250; Abcam, Cambridge, UK) secondary antibodies were used. The sections were mounted on gelatinized slides and were coverslipped. Negative controls were performed by the omission of primary antibody; no staining was observed in these cases.

### 2.9. Immunofluorescence Image Acquisition and Quantification

Single scanned images were acquired by using a confocal Zeiss LSM 510 microscope. To quantify the percentage of cells expressing p47^phox^ in the striatum and SN, we acquired images doubly immunolabeled with antibodies specific to detect different cell types (neurons, astrocytes, and microglial cells) and to detect p47^phox^. A total of eight sampling areas from the control and experimental striatum and SN of each one of the five animals were acquired for analysis. Each slide was scanned at 20x magnification. The red and merged channels from each sampling area were opened in two separate layers of the ImageJ software and compared [21]. By using the ImageJ cell counter plug-in, p47^phox^ immunopositive cells were labeled manually with a colored square, as well as the total number of cells. A ratio was established between the number of p47^phox^ immunolabeled cells and the total amount of cells in the same sampling area, resulting in the percentage of labeled cells. The results were averaged and subjected to statistical analysis using the software GraphPad Prism 3.02. Manipulation of the images was restricted to threshold and brightness adjustments to the whole image.

#### 2.9.1. Semiquantitative Analyses of TH-Immunoreactive Fibers in the Striatum and TH-Immunoreactive Cell Bodies in the SNpc

The numbers of TH-labeled cells in the SNpc were determined by serial section analysis of digital images obtained from stained brain sections using ImageJ (National Institutes of Health, USA). The third section series was sampled, which was supposed to include a representative sample of the SNpc area affected by the striatal 6-OHDA injection. Five sections at a distance of approximately 180 *μ*m from each other were analyzed for the cell counts. Measurements taken from each control and experimental condition from different experimental groups (Wt, gp91phox^−/−^, and Wt treated with apocynin) were averaged and subjected to statistical analysis using the software GraphPad Prism 3.02. The cell loss in the 6-OHDA-lesioned side is expressed as the percentage of the cell number in the control (unlesioned) side.

Striatal TH immunoreactivity was evaluated in terms of optical density. The mean density of neighboring, non-labeled areas in the same sections was used to normalize TH immunoreactivity. The resulting indexes for the control and experimental conditions from different experimental groups (Wt, gp91phox^−/−^, and Wt treated with apocynin) were then compared and subjected to statistical analysis using GraphPad Prism 3.02.

#### 2.9.2. Statistical Analysis

Results are presented as the mean ± standard errors (SEM). Statistical analyses of data were generated using GraphPad Prism, version 3.02 (GraphPad Software Inc., San Diego, CA, USA). For individual comparisons, statistical analysis was performed using unpaired Student's *t*-test. Statistical comparison of more than two groups was performed using analysis of variance (ANOVA), followed by Tukey's test. In all cases, *P* ≤ 0.05 was considered statistically significant.

## 3. Results

### 3.1. NADPH Oxidase Activation

**Figure 1:**
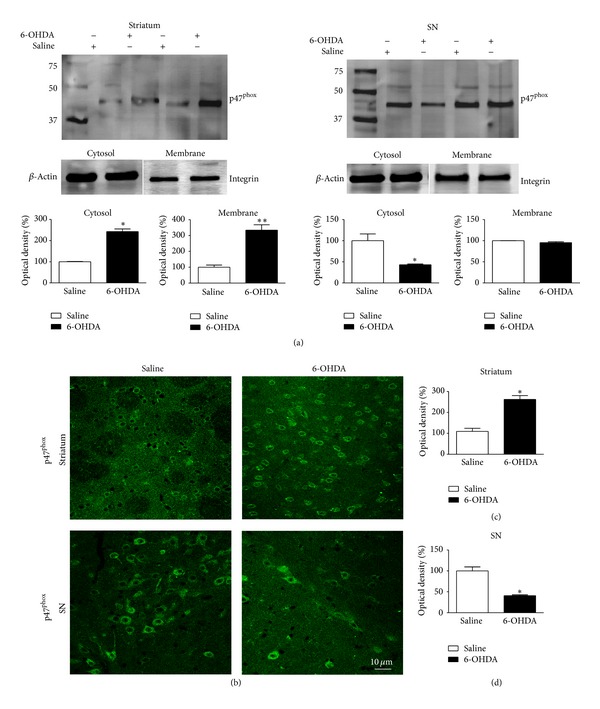
Effect of 6-OHDA on NADPH oxidase activation in SNpc and striatum. (a) representative Western blots illustrating that 6-OHDA stimulates NADPH-oxidase activation, as evidenced by p47^phox^ translocation from the cytosol to the plasma membrane in striatum samples of Wt mice. (b) representative confocal microscopy images illustrating p47^phox^ immunostaining in striatum and SNpc of Wt mice. In (c) and (d), the graphs depict the mean optical density data of 5 samples in each case. *P < 0.05 (Wt saline versus Wt 6-OHDA).

The NADPH oxidase complex includes cytosolic components such as p67^phox^ and p47^phox^ and membrane components [22]. Considering that activation of this enzyme requires the translocation of its cytosolic subunits to the plasma membrane, we first assessed whether 6-OHDA induced increased p47^phox^ subunit in plasma membrane fractions. Fifteen days after 6-OHDA injection, striatum and SN tissue samples were separated into membrane and cytosolic components and examined by Western blotting. As shown in Figure 1, in striatum 6-OHDA induced increased cytosolic and membrane expression of p47^phox^, indicating translocation and activation of the complex, whereas in SN we observed decreased cytosolic protein levels of p47^phox^. Six-OHDA did not change the p47^phox^ membrane protein levels in SN.

**Figure 2:**
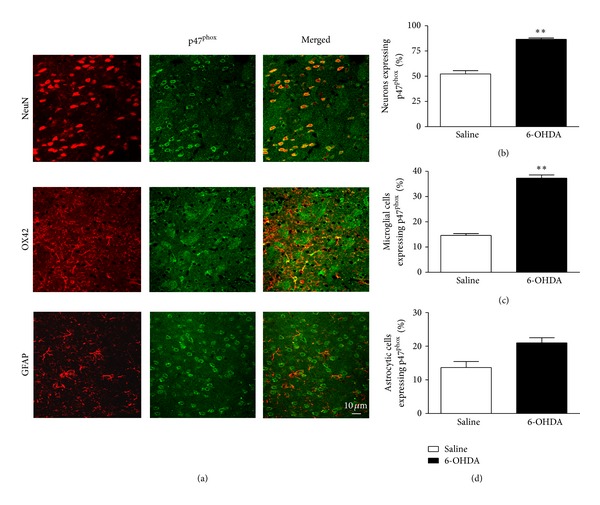
Expression of p47^phox^ in striatum of Wt mice after 6-OHDA injection. (a) representative p47^phox^ immunostaining in neurons and microglia in striatum. Immunostaining was carried out using antibodies for p47^phox^ (shown in green) together with NeuN (top), OX42 (middle), and GFAP (bottom). Microglia, astrocytes, and neuronal markers are shown in red. In striatum, p47^phox^ is expressed in neurons, microglial cells, and rarely with astrocytes. In (b), (c), and (d) the graphs represent the percentage of neurons, microglial cells and astrocytic cells expressing p47^phox^, respectively. **P < 0.01 (Wt saline versus Wt 6-OHDA).

**Figure 3:**
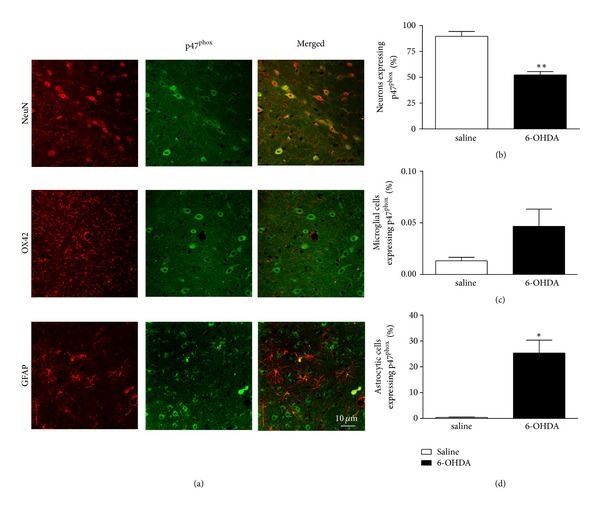
Expression of p47^phox^ in SNpc of Wt mice after 6-OHDA injection. (a) representative p47^phox^ immunostaining in neurons and astrocytic cells. Immunostaining was carried out using antibodies for p47^phox^ (shown in green) together with NeuN (top), OX42 (middle), and GFAP (bottom). Microglia, astrocyte, and neuronal markers are shown in red. In SNpc, p47^phox^-positive cells are basically neurons and astrocytes; however, no p47^phox^-positive cells are OX42-positive cells, thus excluding their microglial origin. In (b), (c), and (d) the graphs represent the percentage of neurons, microglial cells and astrocytic cells expressing p47^phox^, respectively. *P < 0.05 and **P < 0.01 (Wt saline versus Wt 6-OHDA).

To determine the cellular localization of p47^phox^, brain sections containing striatum and SN were examined by double-immunofluorescence staining under confocal microscopy using a p47^phox^ antibody and NeuN, OX42 or GFAP antibodies, markers of neurons, microglial cell, and astrocytes, respectively. In Wt saline-injected mice, slight p47^phox^ immunoreactivity was observed throughout the SN and striatum (Figure 1(b)) and was found to be predominantly located in neurons (data not shown). p47^phox^ immunostaining was found to be strongly increased within striatum of 6-OHDA-injected Wt mice (Figure 1(b)). In agreement with our immunoblotting data, the density of p47^phox^-positive cells was decreased in SN after 6-OHDA injection, possibly due to nigral cell death induced by 6-OHDA (Figure 1(b)). In the striatum of 6-OHDA-injected Wt mice, p47^phox^ was observed in neurons and microglial cells, but not in astrocytes (Figure 2). In the SN, p47^phox^ was found in neurons and in astrocytic cells (Figure 3). These results demonstrate that expression of Nox2 is increased in striatum after 6-OHDA-induced PD, which validates the use of the 6-OHDA as an experimental model to study the involvement of Nox2 in the PD neurodegenerative process.

### 3.2. Apomorphine-Induced Rotation Test

**Figure 4:**
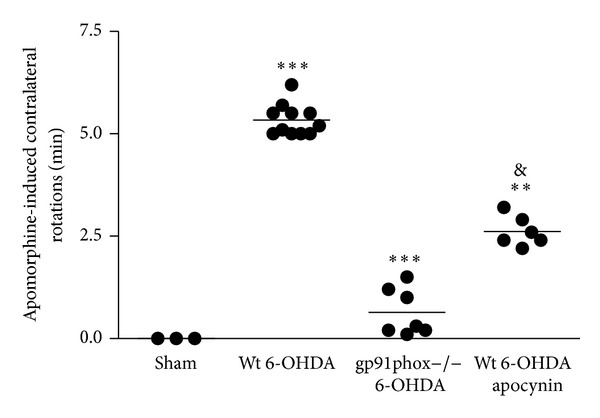
Apomorphine-induced rotation in 6-OHDA-lesioned mice. The graph depicts the number of rotations to the side contralateral to the lesion per minute. ***P < 0.001 sham versus Wt 6-OHDA; ***P < 0.001 Wt 6-OHDA versus gp91^phox−/−^ 6-OHDA; *P* < 0.01 Wt 6-OHDA versus Wt 6-OHDA apocynin; and ^&^ *P* < 0.05  gp91^phox−/−^ 6-OHDA versus Wt 6-OHDA apocynin.

The apomorphine-induced rotation test was performed at day 14 after lesion. Some mice showed stereotyped behaviors, such as sniffing and gnawing after administration of apomorphine. Sham-operated mice exhibited a rotation score of 0.40 ± 0.49, whereas Wt 6-OHDA-lesioned mice showed a mean rotation score towards the control side of 5.33 ± 0.38. Gp91^phox−/−^ 6-OHDA-lesioned mice showed a mean rotation score of 0.87 ± 0.53, whereas 6-OHDA-lesioned Wt mice treated with apocynin had a mean rotation score of 2.61 ± 0.48 (Figure 4).

### 3.3. Semiquantitative Analyses of TH-Immunoreactive Fibers in the Striatum and TH-Immunoreactive Cell Bodies in the SNpc

**Figure 5:**
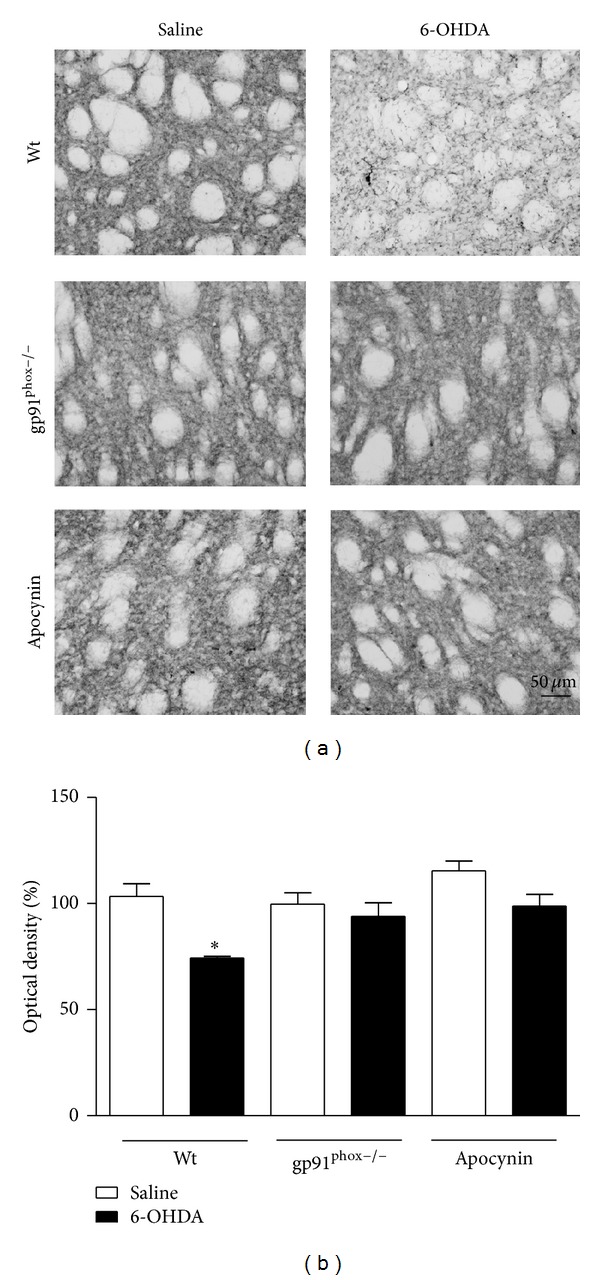
Effects of 6-OHDA on TH immunoreactivity in the striatum. (a) representative digital images of TH immunoreactivity in the saline-injected Wt, 6-OHDA-injected Wt, saline-injected gp91^phox−/−^, 6-OHDA-injected gp91^phox−/−^, apocynin-treated Wt injected with saline, and apocynin-treated Wt injected with 6-OHDA. The graph depicts the mean optical density data of 4 to 5 samples in each case. *P < 0.05 (Wt saline versus Wt 6-OHDA).

**Figure 6:**
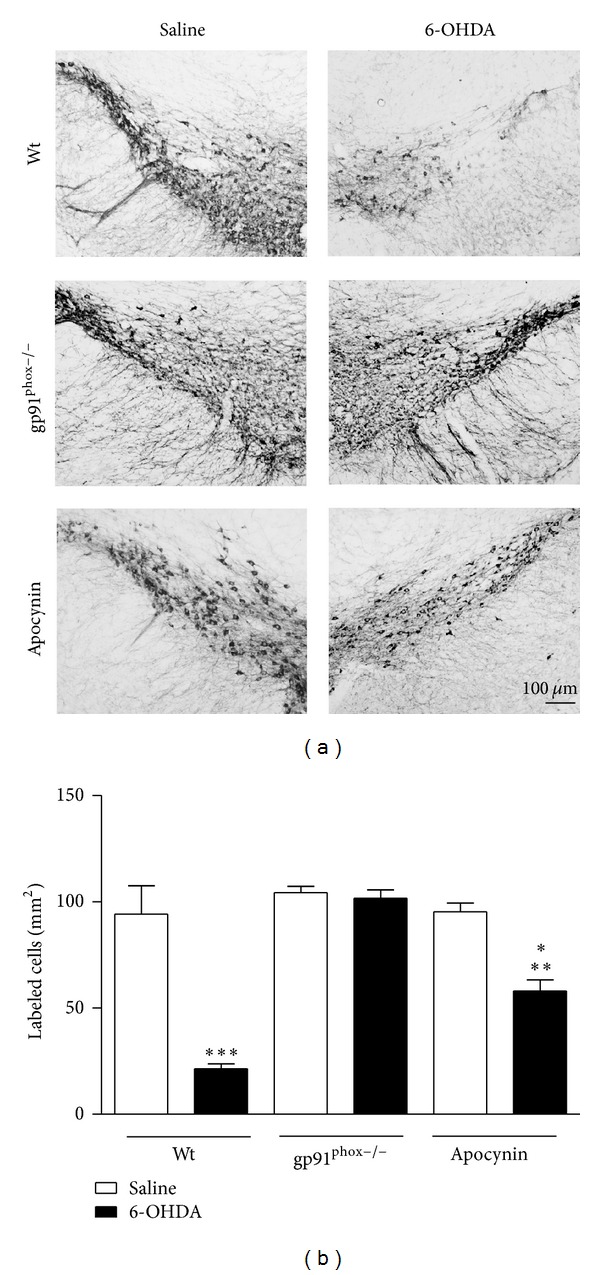
Effects of 6-OHDA on TH immunoreactivity in the SNpc. (a) representative digital images of TH immunoreactivity in mice of the groups: the saline-injected Wt, 6-OHDA-injected Wt, saline-injected gp91^phox−/−^, 6-OHDA-injected gp91^phox−/−^, apocynin-treated Wt injected with saline, and apocynin-treated Wt injected with 6-OHDA. (b) the graph depicts the mean optical density data of 5 to 7 samples in each case. ***P < 0.001 (Wt saline versus Wt 6-OHDA), **P < 0.01 (Wt apocynin saline versus Wt apocynin 6-OHDA), and *P < 0.05 Wt apocynin 6-OHDA versusgp91^phox−/−^ 6-OHDA).

TH-immunolabeling data indicated that the unilateral 6-OHDA injections into the striatum significantly reduced the number of dopaminergic neurons in the SNpc by 82% and of TH staining into the striatum of Wt mice by 29%. Both SNpc and striatal exposure of gp91^phox−/−^ mice to 6-OHDA did not result in significant neuronal loss. Apocynin treatment significantly prevented dopaminergic cell loss in the SNpc (by 39%) and completely recovered TH staining in the striatum (Figures 5 and 6). However, in SNpc, a significant difference between the apocynin-treated and the gp91^phox−/−^ 6-OHDA-lesioned mice groups was found. The treatment with apocynin was able to confer only a limited neuroprotection in comparison with the lesioned gp91^phox−/−^ mice group.

### 3.4. Immunohistochemistry

**Figure 7:**
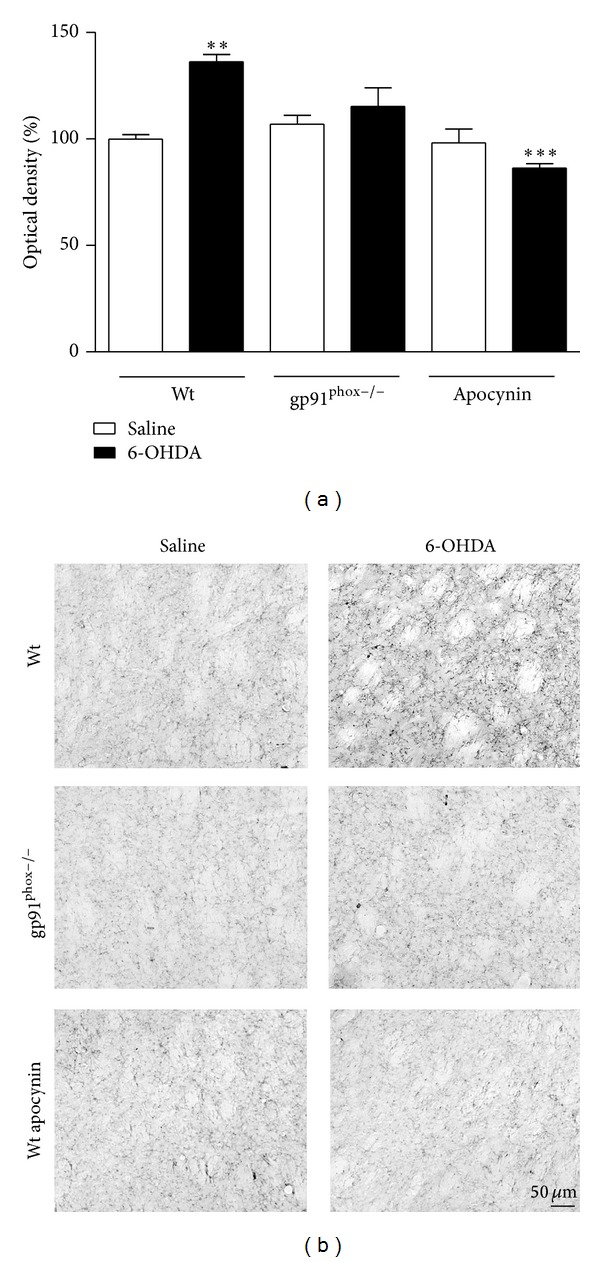
Effects of 6-OHDA on OX42 expression in the striatum. (a) the graph depicts the mean optical density data of 5 to 6 samples in each case. **P < 0.01 (Wt saline versus Wt 6-OHDA) and ***P < 0.001 (Wt 6-OHDAversusWt apocynin 6-OHDA). (b) representative digital images of OX42-like immunoreactivity in the saline-injected Wt, 6-OHDA-injected Wt, saline-injected gp91^phox^, 6-OHDA-injected gp91^phox−/−^, apocynin-treated Wt injected with saline, and apocynin-treated Wt injected with 6-OHDA mice.

**Figure 8:**
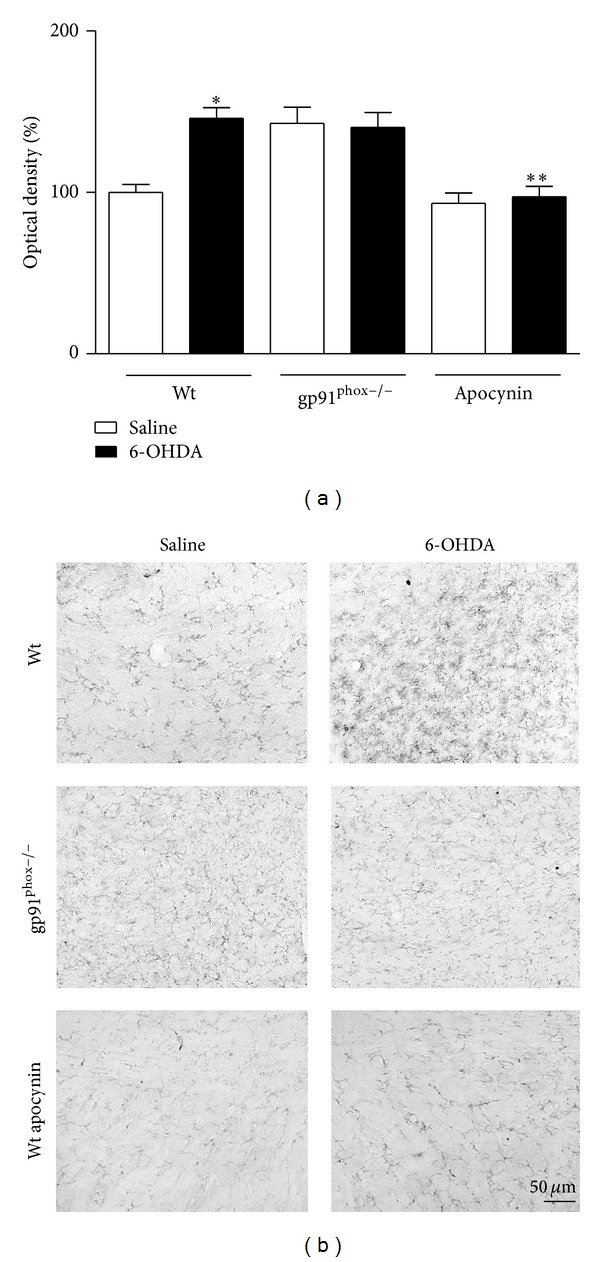
Effects of 6-OHDA on OX42 expression in the SNpc. (a) the graph depicts the mean optical density data of 5 to 6 samples in each case. *P < 0.05 (Wt saline versus Wt 6-OHDA) and **P < 0.01 (Wt 6-OHDA versus Wt apocynin 6-OHDA). (b) representative digital images of OX42-like immunoreactivity in the saline-injected Wt, 6-OHDA-injected Wt, saline-injected gp91^phox^, 6-OHDA-injected gp91^phox−/−^, apocynin-treated Wt injected with saline, and apocynin-treated Wt injected with 6-OHDA mice.

**Figure 9:**
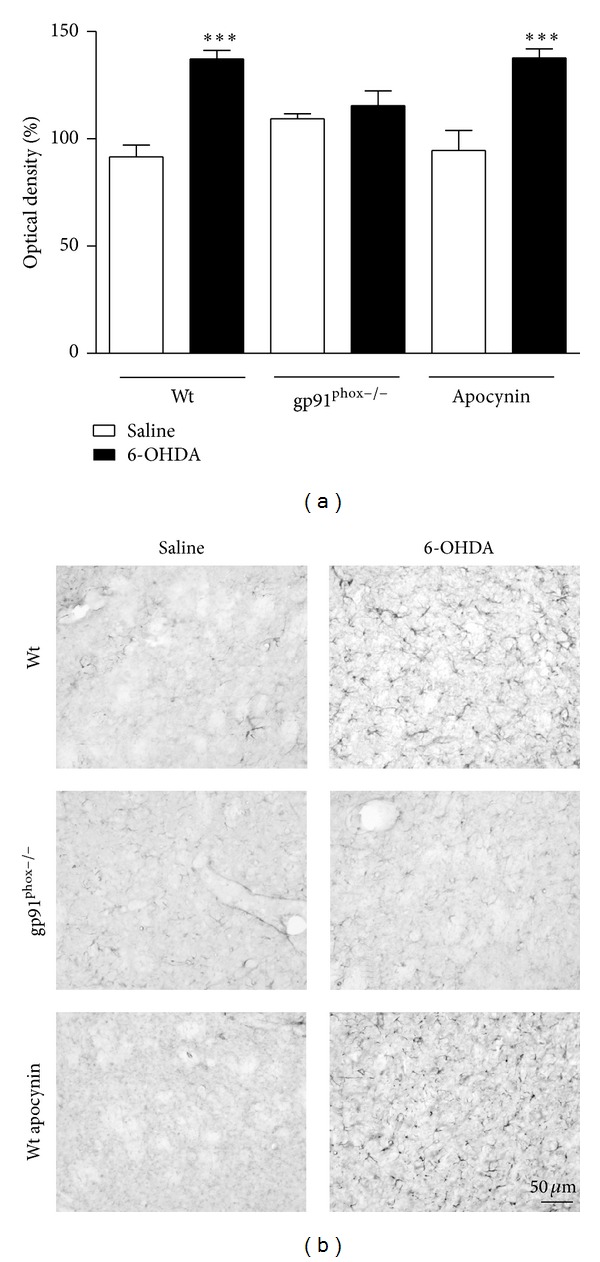
Effects of 6-OHDA on GFAP expression in the striatum. (a) the graph depicts the mean optical density data of 5 to 6 samples in each case. ***P < 0.001 (Wt saline versus Wt 6-OHDA) and ***P < 0.001 (Wt apocynin saline versus Wt apocynin 6-OHDA). (b) representative digital images of GFAP-like immunoreactivity in the saline-injected Wt, 6-OHDA-injected Wt, saline-injected gp91^phox^, 6-OHDA-injected gp91^phox−/−^, apocynin-treated Wt injected with saline, and apocynin-treated Wt injected with 6-OHDA mice.

**Figure 10:**
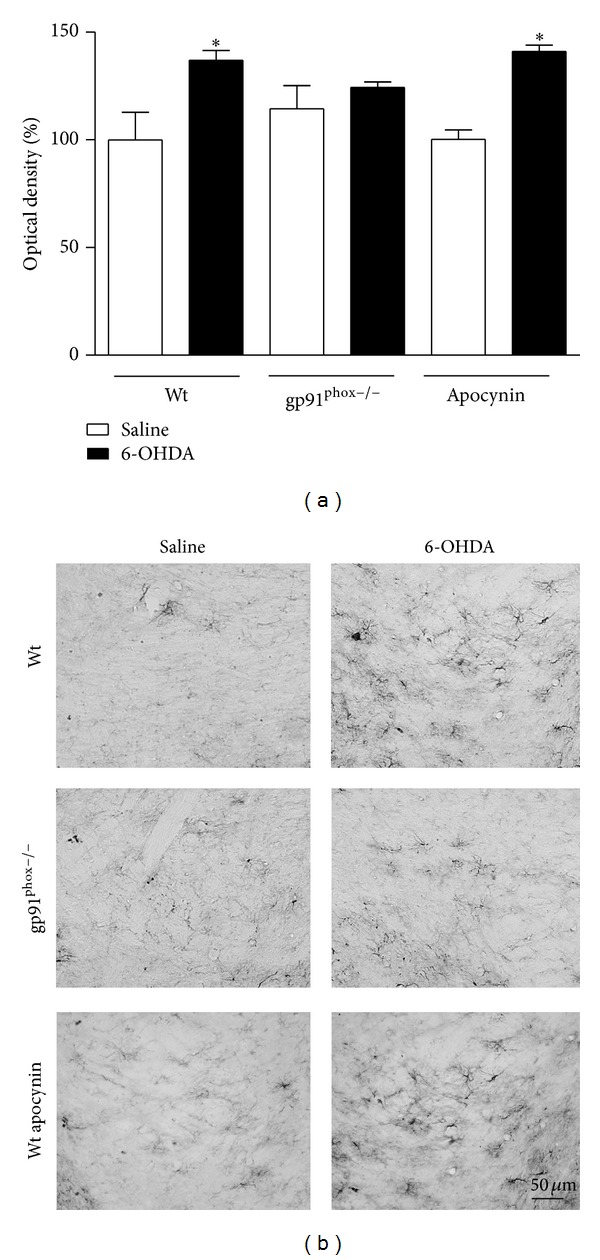
Effects of 6-OHDA on GFAP expression in the SNpc. (a) the graph depicts the mean optical density data of 5 to 6 samples in each case. *P < 0.05 (Wt saline versus Wt 6-OHDA) and *P < 0.05 (Wt apocynin saline versus Wt apocynin 6-OHDA). (b) representative digital images of GFAP-like immunoreactivity in the saline-injected Wt, 6-OHDA-injected Wt, saline-injected gp91^phox^, 6-OHDA-injected gp91^phox−/−^, apocynin-treated Wt injected with saline, and apocynin-treated Wt injected with 6-OHDA mice.

In 6-OHDA-injected Wt mice we observed a marked microglial activation within the striatum (36%) and SNpc (46%) compared to Wt saline-injected mice. In contrast, in gp91^phox−/−^ 6-OHDA-lesioned mice the microglial activation in both structures was negligible when compared to gp91^phox−/−^ saline-injected mice. Apocynin-treated mice showed significantly decreased microglial activation (Figures 7 and 8). Analysis of GFAP immunostaining revealed an increase of GFAP immunostaining into striatum (48%) and SNpc (37%) of Wt 6-OHDA-lesioned mice, which was not reduced by apocynin treatment. In gp91^phox−/−^ mice, 6-OHDA injection did not increase GFAP immunostaining either in the SNpc or in the striatum (Figures 9 and 10).

## 4. Discussion

The Nox family of superoxide and hydrogen peroxide-producing proteins has emerged as an important source of ROS in neurodegenerative conditions [23]. Seven Nox isoforms have been identified, namely, Nox1, Nox2 (also known as gp91^phox^), Nox3, Nox4, Nox5, and dual oxidase 1 and 2 (Duox1 and Duox2), each one with its own specific regulatory subunities and mechanisms. So far, only the presence of Nox1/4 isoforms has been described in the central nervous system (reviewed by [24]). Structurally, all members of the Nox family contain a multisubunit structure, with catalytic flavin-binding Nox subunits and a number of regulatory subunits.

The initial experiments performed here demonstrated that the membrane protein levels of p47^phox^, a cytosolic subunit of the Nox complex, are elevated in striatum of 6-OHDA-lesioned mice, suggesting that this subunit did translocate from the cytosol to the plasma membrane, assembling the functional oxidase capable of producing superoxide. It has been previously demonstrated that 1-methyl-4-phenyl-1,2,3,6-tetrahydropyridine (MPTP) stimulates the translocation of the subunit p67^phox^ from the cytosol to the plasma membrane in the SNpc of Wt mice [25]. The levels of p67^phox^ were also found to be increased in spinal cord extracts from transgenic superoxide dismutase 1 (SOD1) mice, a genetic animal model of amyotrophic lateral sclerosis [26]. Our double immunofluorescence and confocal microscopy assays also revealed that p47^phox^ is expressed in neurons and microglial cells and less often in astrocytes of the striatum. In the SN, p47^phox^ was basically found in neurons. Similar results have also been described in rat mesencephalic cultures 24 hours after the treatment with 6-OHDA [12].

Apocynin is a natural organic compound widely used as a Nox2 activation inhibitor. However, the use of this compound as a specific Nox2 inhibitor remains controversial, since the translocation of the cytosolic subunits is a mechanism required for the activation of at least Nox1 and Nox2 isoforms. Whether apocynin is also able to inhibit the constitutive activity of Nox4 is still a matter of investigation [27].

Despite the apocynin lack of specificity between Nox isoforms, in nonphagocytic cells it has been suggested that this compound acts predominantly as an antioxidant rather than a Nox inhibitor [27]. Apocynin is activated by myeloperoxidases or H_2_O_2_, resulting in the formation of a covalent apocynin dimer, known as diapocynin. Apocynin dimers are able to inhibit the Nox complex by oxidizing thiols of the subunit p47^phox^. Thus, the inhibitory effect of apocynin on Noxes seems to be restricted to cells that produce H_2_O_2_ and/or express myeloperoxidases. Endothelial cells and smooth muscle cells, for example, failed to produce diapocynin *in vitro* [27]. In the nervous system, following a single intraperitoneal injection of apocynin (5 mg/kg), no diapocynin was detected in brain samples of rats within 30 min to 2 hours after administration [28]. On the other hand, another study has shown that after the chronic treatment with apocynin (150 mg/kg in the drinking water for 100 days), micromolar concentrations of diapocynin were found accumulated in the brain tissue of transgenic SOD1 mutant mice. Its content represented about 7-8% of the total apocynin concentration [29]. Although there are some experimental evidence describing the neuroprotective effects of apocynin under different models of PD [12, 30, 31], more detailed studies elucidating the production of diapocynin in brain structures affected by this neurodegenerative condition are needed to elucidate whether these promising findings are related to Nox inhibition rather than apocynin antioxidant effects. Considering the apocynin lack of specificity and elusive mechanism of action, in the present study, we sought to compare the effect of 6-OHDA-induced PD in mutant mice lacking gp91^phox^ and by the pharmacological inhibition of the Nox complex with apocynin.

Since unilateral 6-OHDA injection causes a quantifiable rotational behavior induced by systemic administration of dopaminergic receptor agonists such as apomorphine [32], we further analyzed the extent of the dopaminergic lesion induced by this neurotoxin. We found in the present study that both gp91^phox−/−^ 6-OHDA-lesioned mice and Wt 6-OHDA-lesioned mice chronically treated with apocynin exhibited significantly ameliorated neurotoxin-induced rotational behavior in comparison with Wt mice. However, the expression of contralateral turnings after systemic injections of apomorphine between gp91^phox−/−^ 6-OHDA-lesioned mice and Wt 6-OHDA-lesioned mice chronically treated with apocynin was found to be different. Gp91^phox−/−^ presented a significantly reduced rotation score when compared to the apocynin-treated mice group.

We also found a positive relationship between apomorphine-induced rotational behavior and TH-immunoreactive cell bodies and fibers. We confirmed that the administration of apocynin was able to attenuate dopaminergic neurodegeneration induced by 6-OHDA in SNpc and from the dopaminergic terminal loss in striatum. However, gp91^phox−/−^ mice appear to be completely protected from the dopaminergic cell loss induced by 6-OHDA in the SNpc, an effect significantly different from the effect observed in the apocynin-treated group. Regarding the use of gp91^phox−/−^ mice, our results are in accordance with an earlier study in which it was demonstrated that these knockout mice exhibited less SNpc dopaminergic neuronal losses and protein oxidation than Wt mice after MPTP injections [25]. Furthermore, primary mesencephalic cultures from gp91^phox−/−^ mice were more resistant to rotenone neurotoxicity than those from Wt mice [30].

It has been previously demonstrated that, in addition to the dramatic loss of dopaminergic neurons, gliosis is a marked neuropathological feature in the SNpc and the striatum in the 6-OHDA-mouse models of PD [33–35]. In mesencephalic primary cultures, activated microglia generated Nox-derived superoxide and enhanced neurotoxin-elicited dopaminergic neurodegeneration [30]. Furthermore, it was reported that inhibition of microglial activation can protect dopaminergic neurons from degeneration [35, 36]. Immunohistochemical detection of OX42-positive cells in this study indicated a marked microglial activation in both striatum and SNpc of mice injected with 6-OHDA. Our immunostaining assays also revealed increased expression of astrocyte markers in the striatum and SNpc after 6-OHDA injection. Our data show that gp91^phox−/−^ mice are protected against glial activation induced by 6-OHDA, whereas apocynin treatment was only able to prevent microglial activation.

## 5. Conclusions

In summary, our data emphasize that the Nox2-dependent oxidative stress contributes to PD-dopaminergic neurodegeneration in the nigrostriatal pathway. In addition, the experiments conducted with apocynin and mutant mice deficient in gp91^phox^ collectively demonstrate no comparable 6-OHDA effects between the two mice groups. The apocynin treatment conferred only a limited protection. As an alternative to the use of unspecific Nox inhibitors, the use of knockout mice may represent several advantages to characterize the individual contribution of ROS producing systems such as Noxes.
